# Por que Desenvolvemos Modelos – Da Prática Clínica de Cardiologia a Epidemias de Doenças Infecciosas

**DOI:** 10.36660/abc.20200527

**Published:** 2020-06-29

**Authors:** Marcio Sommer Bittencourt

**Affiliations:** 1 Centro de Medicina Preventiva Hospital Israelita Albert Einstein Faculdade Israelita de Ciência da Saúde Albert Einstein São PauloSP Brasil Centro de Medicina Preventiva, Hospital Israelita Albert Einstein e Faculdade Israelita de Ciência da Saúde Albert Einstein, São Paulo, SP - Brasil; 2 Centro de Pesquisa Clínica e Epidemiológica Hospital Universitário Universidade de São Paulo São PauloSP Brasil Centro de Pesquisa Clínica e Epidemiológica, Hospital Universitário, Universidade de São Paulo, São Paulo, SP - Brasil; 3 Diagnósticos da América São PauloSP Brasil Diagnósticos da América (DASA), São Paulo, SP - Brasil

**Keywords:** Doenças Cardiovasculares, Biomarcadores, Fatores de Risco, Medição de Risco, Comportamento de Redução de Risco, Prevenção e Controle, Coronavirus, COVID-19, Pandemia

Francisco, 64 anos, vem ao seu consultório para uma avaliação preventiva. Ele tem um histórico de hipertensão bem controlada e não tem nenhuma queixa. Não tem outras comorbidades ou doenças prévias. Não há histórico familiar de doença cardiovascular ou tabagismo e LDL-colesterol (LDL-C) é de 90 mg/dL. Depois de discutir com o paciente, você não tem certeza se o perfil de benefício de risco desse paciente justificaria uso de estatina. Em vez de confiar em sua sensação subjetiva, você decide usar o escore de risco de Framingham (FRS) para definir o uso de estatina.^1^ Como o risco calculado pelo FRS foi de 8,1%, você decide não iniciar estatina neste momento.

Um mês depois, Francisco retorna com angina típica aos grandes esforços, mas sem sinais de instabilidade. Mais uma vez para evitar excesso de confiança apenas em sua impressão inicial, você decide usar a escala de predição de dor torácica Diamond e Forrester (DF), que estima a probabilidade pré-teste de doença arterial coronariana obstrutiva (DAC).^2^ Para um homem na idade dele, a regra sugere uma probabilidade pré-teste de 94% (alta probabilidade) e você decide solicitar uma cineangiocoronariografia invasiva.

Antes de realizar o exame, Francisco apresenta piora da dor torácica mesmo em repouso. O paciente procura o pronto-socorro. A dosagem de troponinas está normal e o ECG em repouso tem infradesinvelamento do segmento ST de 1 mm nas derivações anteriores. O escore de risco de TIMI é de 1, indicando baixo risco.^3^ O paciente é internado por 48 horas, passa por um teste de esteira negativo limitado pela baixa capacidade física, mas recebe alta para casa com a medicação apropriada. Uma semana depois, ele retorna ao hospital com infarto do miocárdio com supra de ST. Ele é levado para a angioplastia primária de lesão grave em terço médio de artéria coronária média direita. Depois de três dias no hospital, ele tem alta.

Os escores de FRS, DF ou TIMI foram corretos ou incorretos na estratificação de risco? Eles conseguiram prever o que aconteceu com o paciente? O FRS estratificou o paciente como baixo risco, <10% de risco de um evento cardiovascular maior em 10 anos. O DF sugeriu presença quase certa de DAC obstrutiva, enquanto o escore de risco de TIMI sugeriu baixo (5%) risco de morte, infarto agudo do miocárdio (IAM) recorrente ou isquemia grave em duas semanas. No entanto, o paciente apresentou IAM menos de uma semana depois.

Os cardiologistas são acostumados a utilizar escores de risco derivados de modelos de predição. Os modelos são simplificações da vida real, o que os torna mais generalizáveis para uma população mais ampla e externamente válidos para outros indivíduos além da coorte inicial de pacientes onde os escores foram desenvolvidos. Esses modelos selecionam um número limitado de variáveis consideradas de maior importância para prever o desfecho desejado. No entanto, várias premissas são feitas para cada variável e para a população a que se aplicam. Se tais suposições mudarem, o modelo pode não ser mais válido ou pode precisar ser recalculado ou recalibrado para se adequar ao novo ambiente. Por exemplo, o FRS considera apenas as condições de fumante ou não fumante. Assim, ex-fumantes são considerados de risco semelhante aos não fumantes, enquanto indivíduos que fumam dois cigarros por dia são considerados de risco semelhante aos que fumam três maços por dia. Esses aspectos podem levar a imprecisão na estimativa individual de risco. No entanto, eles podem ter implicações muito maiores quando tais mudanças no valor de cada variável ocorrem em nível populacional. Por exemplo, quando o FRS foi derivado, o fumante médio fumava de um a dois maços por dia. Atualmente, a maioria dos fumantes fuma menos de um quarto disso. Assim, a aplicação da versão antiga do FRS na atualidade pode resultar em uma estimativa de risco incorreta.

Essas dificuldades no uso de modelos de predição são conhecidas pelo cardiologista. Inclusive, a maioria dos escores são eventualmente atualizados para aumentar a precisão com o uso de variáveis novas ou recalibradas. Usando o exemplo de caso acima, pode-se sugerir o escore americano de doença cardiovascular aterosclerótica ( *atherosclerotic cardiovascular disease* — ASCVD) em vez do FRS. Utilizando o escore ASCVD, o risco de eventos cardiovasculares maiores em 10 anos seria de 10,6% e, de acordo com as novas diretrizes, seria recomendado o uso de estatina para reduzir o risco cardiovascular desse paciente.^4^ Espera-se que esse modelo atualizado seja mais preciso para predizer risco.

Em outras situações, pode-se até considerar que o modelo não seja mais útil e toda a abordagem deva ser diferente. Por exemplo, pode-se dizer que um infradesnivelamento do segmento ST de 1 mm em repouso configuraria alto risco independente de outras características clínicas. Nesse caso, o paciente teria sido encaminhado para cineangiocoronariografia invasiva durante a primeira internação e poderia não ter apresentado o IAM subsequente.

Finalmente, se o paciente tivesse realizado a cineangiocoronariografia antes de apresentar instabilização, ele poderia ter sido adequadamente tratado com aspirina, estatinas, betabloqueadores e outras medicações. Nesse cenário, esse paciente poderia ter vivido mais 10 anos sem qualquer outra complicação cardiovascular. Nesse caso, consideraríamos o seu FRS inicial como certo ou errado?

Os modelos não devem ser avaliados tardiamente para serem julgados como certos ou errados. A questão correta é se o modelo foi adequadamente desenhado para a situação em que está sendo utilizado, se o resultado que pretende prever é de interesse e se as informações fornecidas são incrementais ao que é conhecido no momento em que o modelo é utilizado. Quando tais premissas são atendidas, os modelos podem levar a decisões mais bem informadas que podem ter impacto significativo. No caso acima, o uso adequado do escore ASCVD na apresentação inicial ou uma interpretação diferente do infradesnivelamento do segmento ST poderia ter levado a mudanças no tratamento que poderiam alterar completamente a história da doença desse paciente.

Embora os cardiologistas clínicos mais experientes não se surpreendam com tais peculiaridades da modelagem da predição de risco, elas nem sempre são bem compreendidas pelo público leigo. Um problema semelhante é agora observado com destaque dado aos modelos epidemiológicos para a previsão do surto de COVID-19. Um modelo inicial publicado pelo Imperial College London sugeriu que o surto poderia ter grande impacto em todo o mundo,^5^ com mortes relacionadas à COVID-10 atingindo milhões nos Estados Unidos e no Reino Unido. O modelo também estimou o impacto de possíveis intervenções para controlar o surto que poderiam levar a uma redução colossal nas mortes. Outros modelos se seguiram, com números muito mais baixos, às vezes com ordens de magnitude inferiores aos cenários anteriores. Isso levou diversas vozes da comunidade científica, imprensa leiga e público em geral a fazer críticas incisivas contra esses modelos iniciais. A maior parte delas usando dados atuais ou projeções mais novas para ilustrar o quão “errado” o modelo inicial estava.

O desenvolvimento de modelos epidemiológicos para a COVID-19 tem pouca semelhança com os modelos mais simples utilizados para a previsão de risco em cardiologia, mas ambos utilizam dados atuais e prévios para projetar um cenário futuro e tentar estimar o valor das intervenções para reduzir o risco de desfechos negativos. No entanto, devido ao tempo limitado desde que a COVID-19 foi descoberta, vários parâmetros relacionados ao comportamento do vírus são estimados com base em dados preliminares bastante restritos. Às vezes, quando não há dados disponíveis, os parâmetros são apenas estimativas imprecisas baseadas em outras doenças ou condições semelhantes mais conhecidas. Além disso, esses modelos dependem da transmissão viral, um processo complexo que pode envolver parâmetros difíceis de estimar, como o número médio de interações sociais que cada indivíduo tem ou a densidade demográfica em cada área. Algumas dessas informações não estão facilmente disponíveis, e mais uma vez uma estimativa imprecisa pode ser utilizada pelos pesquisadores. Um exemplo é o uso de dados do Peru em um dos modelos do ICL para o cenário brasileiro para uma variável em que dados locais não estavam disponíveis para o Brasil. Com variáveis tão limitadas, não surpreende que tais modelos tenham grande variabilidade.

No entanto, isso é apenas uma parte da questão ao interpretar modelos após surto. Embora mudanças específicas nas intervenções possam ser consideradas no modelo, é impossível “prever” como o governo ou a população se comportarão no futuro, assim como não se pode prever se o paciente começará a fumar quando o risco cardiovascular é inicialmente calculado, como no caso inicial. Mesmo que o distanciamento fisico seja considerado no modelo, seu verdadeiro impacto depende do quanto a população segue tais medidas. Por exemplo, embora medidas rigorosas para aumentar o distanciamento social tenham sido propostas para a cidade de São Paulo, o governo reconhece que elas não alcançaram mais da metade do efeito esperado. Logo, sabe-se que seu impacto também será menor.

No entanto, mesmo que os modelos sejam bem sucedidos, eles podem ser interpretados como incorretos no futuro. Por exemplo, o modelo supracitado do ICL apresentou um cenário tão catastrófico que levou a mudanças em políticas públicas substanciais em todo o mundo. Se essas mudanças levarem a uma redução da taxa de mortalidade devido a sua implementação precoce e eficaz, tal redução nas mortes poderia levar a afirmações de que o modelo estava “errado” pois tinha estimado muito mais mortes.

Outro aspecto importante dos modelos durante uma epidemia como a COVID-19 é que quanto mais cedo eles são criados, menos informações estão disponíveis, levando a um modelo menos preciso. No entanto, quanto mais cedo o modelo for desenvolvido, maior será o impacto das intervenções derivadas dele. Em um mundo de informações perfeitas, a COVID-19 poderia ter sido extinta se as informações que temos atualmente sobre a doença estivessem disponíveis quando o primeiro caso foi diagnosticado e ele e seus contatos tivessem sido isolados desde o princípio. Por outro lado, o conhecimento perfeito de todos os detalhes da transmissão e propagação viral seriam de pouco impacto social depois que o surto terminasse. Assim, resta-nos conviver com as incertezas e imprecisões derivadas dos modelos e esperamos que tais modelos apareçam em tempo de orientar intervenções políticas eficazes.

Assim, para termos modelos bem sucedidos, precisamos aceitar, entender e reconhecer tais limitações. Além disso, precisamos ser humildes para ajustar as velas às condições do vento e atualizar e melhorar nossos modelos ao longo do caminho. Cada modelo só deve ser julgado tendo em conta o momento em que foi desenvolvido, incluindo as limitações do conhecimento disponível na época. No final, seria como para o caso do Sr. Francisco: poderíamos ter melhorado a previsão inicial de risco e a história de sua vida com um modelo inicial melhor para estimar seu risco cardiovascular. No entanto, após seu infarto do miocárdio, mesmo a informação perfeita da estratificação de seu risco cardiovascular seria de pouco valor. Assim como na prática clínica, ao avaliar esses modelos epidemiológicos, devemos abster-nos de ser médicos do dia seguinte, que estão sempre certos depois que o diagnóstico já é conhecido. Em vez de apontar agressivamente os dedos para modelos que sabemos ser incertos, sejamos humildes e práticos ao avaliá-los. O modelo foi capaz de informar melhor as intervenções na sua época, e foi capaz de reduzir, mesmo que por pouco, as imprecisões que tínhamos? Se sim, então o modelo foi útil, mesmo que estivesse errado.
