# Supplementary material for: Por que Desenvolvemos Modelos – Da Prática Clínica de Cardiologia a Epidemias de Doenças Infecciosas
Source: Arq Bras Cardiol. 2020 Jun 29;114(6):992–4. [Article in Portuguese] doi: 10.36660/abc.20200527 (PMC8416127; doi:10.36660/abc.20200527)
Supplement: Translation

# Translation: Why We Build Models – From Clinical Cardiology Practice to Infectious Disease Epidemics

**Authors:** Marcio Sommer Bittencourt

**Affiliations:** 1 Brazil Centro de Medicina Preventiva, Hospital Israelita Albert Einstein e Faculdade Israelita de Ciência da Saúde Albert Einstein, São Paulo, SP - Brazil; 2 Brazil Centro de Pesquisa Clínica e Epidemiológica, Hospital Universitário, Universidade de São Paulo, São Paulo, SP - Brazil; 3 Brazil Diagnósticos da América (DASA), São Paulo, SP - Brazil

**Keywords:** Cardiovascular Diseases, Biomarkers, Risk Factors, Risk Assessment, Risk Reduction Behavior, Prevention and Control, Coronavirus, COVID-19, Pandemics

Francisco, 64 years old, comes to your office for a preventive health evaluation. He has a history of well-controlled hypertension and is otherwise well. His past medical history is unremarkable. No family history of cardiovascular disease or smoking and LDL-cholesterol (LDL-C) of 90 mg/dL. After discussing with the patient, you are unsure if this patient’s risk benefit profile would favor the use of statins. Instead of trusting your personal feelings, you decide to use the Framingham risk score (FRS) to decide if statins would be recommended.^1^ The calculated Framingham score is 8.1% and you decide not to initiate statins at this point.

One month later, Francisco returns to your office with typical angina on major exertion but has no signs of unstable disease. Once again, to avoid overconfidence on your initial impression, you decide to use the Diamond and Forrester (DF) chest pain prediction rule, which estimates the pretest probability of obstructive coronary artery disease (CAD).^2^ For a male at his age, the rule suggests a pretest probability of 94% (high probability), so you decide to request an invasive angiography in the outpatient setting.

A couple of days before the test, Francisco calls you complaining of worsening chest pain similar to the previous presentation, but now at rest. You tell the patient to go to the emergency room, where troponins are normal and resting ECG has 1 mm ST-segment depression in the inferior leads. Calculated TIMI risk score is 1, indicating low risk.^3^ The patient is admitted for 48 hours, undergoes a negative treadmill test limited by poor physical performance and is discharged home with appropriate medication. One week later, he returns to the hospital with ST-segment elevation myocardial infarction. He is rushed to the Cath lab and a severe lesion in the mid right coronary artery is documented. He undergoes a percutaneous coronary intervention. After three days in the hospital, he is discharged home.

Were the FRS, the DF or the TIMI risk scores correct or incorrect? Were the scores able to predict what happened to the patient? The FRS stratified the patient as low risk, meaning less than 10% risk of a major cardiovascular event in 10 years. The DF suggested an almost certain presence of obstructive CAD, whereas the TIMI risk score suggested low (5%) risk of death, recurrent myocardial infarction (MI) or severe ischemia requiring urgent revascularization in two weeks. Yet, the patient presented MI less than one week later.

Cardiologists are used to risk scores derived from risk prediction models. Models are simplifications of real life, and such simplifications make models generalizable to a broader population and externally valid to other individuals beyond the initial cohort of patients where they were developed. The models select a limited number of variables considered of higher importance to predict the desired outcome, but several assumptions are made based on each of those variables and the population they apply to. If such assumptions change, the model may no longer be valid or it might need to be recalculated or recalibrated to fit the new environment. For example, the FRS considers smoking as present or absent. Thus, past smokers are considered of similar risk to non-smokers. Also, individuals smoking two cigarettes a day are considered just like those smoking three packs a day. While those aspects may lead to imprecision when estimating the risk of an individual, they can have much broader implications when such changes in the value of each parameter occur at the population level. For example, when the FRS was derived, an average smoker would smoke one to two packs a day. Currently, most smokers smoke less than a quarter than that. Thus, applying the old version of the FRS in the current reality might result in an incorrect risk estimation.

This issues with models are known to cardiologists, and most scores are, at some point, updated to recalibrate and improve precision using new or recalibrated variables. Using the example case above, one might suggest the use of the atherosclerotic cardiovascular disease (ASCVD) score instead of the FRS. Using the ASCVD score, the 10-year risk of major cardiovascular events would be 10.6% and, according to newer guidelines, statins would be recommended to reduce this patient’s cardiovascular risk.^4^ This updated risk stratification score is expected to be more accurate.

In other situations, we may even consider the model as no longer useful and the entire approach should be different. For example, one could say 1 mm ST-segment depression at rest would characterize high risk irrespective of other clinical characteristics. In such case, the patient would have been sent to the Cath lab upon the initial presentation and would not have had MI.

Finally, if the patient made into the Cath lab before clinical worsening, he could have been adequately managed medically with aspirin, statins, betablockers and other therapies. In such scenario, this patient could have lived another 10 years without any other clinical manifestation of ASCVD. Would his initial FRS be then interpreted as right or wrong?

Models should not be judged right or wrong long after they are developed. The appropriate question is whether the model had been adequately designed to the situation where it is being used, whether the outcome it aims to predict is of interest and whether the information is incremental to what is currently known. When such premises are met, models can lead to more well-informed decisions that may have a meaningful impact. In the case above, the adequate use of the ASCVD score in the initial presentation or a different interpretation of the ST-segment depression could have led to changes in management and completely modify the history of the disease for this patient.

While even the most experienced clinical cardiologists are unsurprised by such peculiarities of risk prediction models, they are not always well understood by lay people. A similar problem is now seen with the raising relevance assigned to epidemiological models for the prediction of the COVID-19 outbreak. An initial model published by the Imperial College London suggested that the outbreak could have a major impact across the world,^5^leading to millions of deaths related to COVID-10 in the United States and the United Kingdom. The model also estimated the impact of potential interventions leading to a colossal reduction in deaths. Other models followed, with much lower numbers, sometimes orders of magnitude lower than prior worst-case scenarios leading to several voices in the scientific community, the lay press and the general public to raise strong criticism against those initial models, most of which using current or newer projections to illustrate how “wrong” the initial model was.

The development of epidemiological models for COVID-19 have little resemblance to the simpler models used for risk prediction in cardiology. Yet, both use current and prior data to project a future scenario trying to estimate the value of interventions to reduce the risk of negative outcomes. However, due to the limited time since COVID-19 was discovered, several parameters related to the behavior of the virus are estimated based on restricted preliminary data. Sometimes, when no data is available, parameters are only best guesses based on related conditions or prior comparable situations. Additionally, such models are dependent on the viral transmission, a complex process that may involve hard-to-estimate parameters, such as the average number of social interactions each individual has or demographic density in each area. Some of those inputs might not be available and, once again, the best-informed guess is used by modelers. An example is the use of some data from Peru in an ICL model for the Brazilian case for pieces of data that were unavailable for Brazil. With such limited data inputs, it should come as no surprise that such models include immense variability.

Yet, this is only part of the issue when interpreting post-outbreak models. Although specific changes in the interventions can be considered in the model, it is impossible to predict how the government or the population will behave in the future, just like one cannot predict if the patient will start smoking when the cardiovascular risk is initially calculated. Even if social distancing is considered in the model, its true impact depends on how much the population follows such measures. For example, while strict measures to increase social distancing had been proposed for the city of São Paulo, the government recognizes they did not achieve more than half of the expected effect. Thus, its impact is also expected to be lower.

Yet, even if models are successful, they might be interpreted as being wrong in the future. For example, the aforementioned model from the ICL presented such a catastrophic scenario that it led to substantial policy changes across the world. If those changes led to a substantially lower death rate due to its early and effective implementations, such reduction in deaths could lead to claims that the model was “wrong” because it overestimated deaths.

Another important aspect of models in an epidemic such as COVID-19 is that the earlier they are developed, the less information is available, leading to a less precise model. However, the earlier the model is developed, the larger the impact of interventions derived from it. In a world of perfect information, COVID-19 could have been extinct if the information we currently have were available when the first case was diagnosed and the first case and its contacts were isolated. On the other extreme, perfect details of transmission and viral spread would be of little social impact after the outbreak ended. Hence, we are left to live with the uncertainty and imprecisions derived from models, particularly if we expect that such models will appear in time to guide effective policy interventions.

Thus, to have successful models, we need to accept, understand and acknowledge such limitations. Additionally, we need to be humble enough to adjust the sails to the ever-changing wind conditions update and improve our models along the way. Each model should only be judged bearing in mind the time when it was developed, including the limited knowledge available back then. In the end, it would be just like Francisco’s case: we could have improved the initial risk prediction and the course of his life with a better initial model to estimate his cardiovascular risk. Yet, after his myocardial infarction, even perfect information on his cardiovascular risk would be of little value. Just like in clinical medicine, when those epidemiological models are evaluated, we should refrain from being next day’s doctors who are always right after the diagnosis is known. Instead of aggressively pointing fingers at models that are known to be uncertain, let us be humble and practical when evaluating them. Was the model able to better inform interventions at its time and was it able to reduce, even by a little, the imprecisions we had? If yes, then the models were useful, even if wrong.
